# A Comprehensive Engineering Analysis of Left Heart Dynamics After MitraClip in a Functional Mitral Regurgitation Patient

**DOI:** 10.3389/fphys.2020.00432

**Published:** 2020-05-07

**Authors:** Andrés Caballero, Wenbin Mao, Raymond McKay, Rebecca T. Hahn, Wei Sun

**Affiliations:** ^1^Tissue Mechanics Laboratory, The Wallace H. Coulter Department of Biomedical Engineering, Georgia Institute of Technology and Emory University, Atlanta, GA, United States; ^2^Division of Cardiology, The Hartford Hospital, Hartford, CT, United States; ^3^Division of Cardiology, Columbia University Medical Center, New York, NY, United States

**Keywords:** mitral valve, MitraClip, fluid-structure interaction, functional mitral regurgitation, heart failure, patient-specific, edge-to-edge, annuloplasty

## Abstract

Percutaneous edge-to-edge mitral valve (MV) repair using MitraClip has been recently established as a treatment option for patients with heart failure and functional mitral regurgitation (MR), which significantly expands the number of patients that can be treated with this device. This study aimed to quantify the morphologic, hemodynamic and structural changes, and evaluate the biomechanical interaction between the MitraClip and the left heart (LH) complex of a heart failure patient with functional MR using a fluid-structure interaction (FSI) modeling framework. MitraClip implantation using lateral, central and double clip positions, as well as combined annuloplasty procedures were simulated in a patient-specific LH model that integrates detailed anatomic structures, incorporates age- and gender-matched non-linear elastic material properties, and accounts for mitral chordae tethering. Our results showed that antero-posterior distance, mitral annulus spherecity index, anatomic regurgitant orifice area, and anatomic opening orifice area decreased by up to 28, 39, 52, and 71%, respectively, when compared to the pre-clip model. MitraClip implantation immediately decreased the MR severity and improved the hemodynamic profile, but imposed a non-physiologic configuration and loading on the mitral apparatus, with anterior and posterior leaflet stress significantly increasing up to 210 and 145% during diastole, respectively. For this patient case, while implanting a combined central clip and ring resulted in the highest reduction in the regurgitant volume (46%), this configuration also led to mitral stenosis. Patient-specific computer simulations as used here can be a powerful tool to examine the complex device-host biomechanical interaction, and may be useful to guide device positioning for potential favorable clinical outcomes.

## Introduction

The mitral valve (MV) repair technique using MitraClip (Abbott, Santa Clara, CA, United States) is the most common percutaneous treatment option for patients with symptomatic mitral regurgitation (MR) at high surgical risk (Mauri et al., 2013). Although MitraClip was recently approved by the U.S. Food and Drug Administration (FDA) for use in heart failure patients with functional MR, two large randomized clinical trials showed apparently conflicting results. While the COAPT trial showed that MitraClip was associated with a lower rate of hospitalization for heart failure and lower all-cause mortality within 24 months of follow-up, compared with using medical therapy alone (Stone et al., 2018), the MITRA-FR trial did not show significant differences between the intervention and control groups (Obadia et al., 2018).

Differences in the two clinical trials are likely to be related to threshold values for MR severity, medical management, operator experience and most importantly, proper patient selection (Goldberg, 2019); with markedly improved outcomes in the setting of a disproportionately larger severity of MR relative to left ventricle (LV) volumes after optimizing medical therapy (Grayburn et al., 2019). Indeed, several studies have shown that persistence of moderate-to-severe MR after MitraClip is associated with a considerably higher 1-year mortality (Lim et al., 2014; Sorajja et al., 2017; Ailawadi et al., 2019), such that MR reduction to moderate or less is of paramount importance. Thus, it is clear that for a successful MitraClip therapy: (i) a multidisciplinary heart team needs to be involved, (ii) procedural techniques need to be optimized, (iii) a better mechanistic understanding of device-host interaction is needed, and (iv) physicians need to perform careful patient selection and individualize treatments in accordance with patient characteristics.

To support this multidisciplinary approach, patient-specific computer simulations for transcatheter cardiac interventions can help to better understand the complex biomechanical inter-dependence between the device and the human host, predict device performance (efficacy), and possible complications (safety) (de Jaegere et al., 2019). Computer simulations using finite element (FE) and fluid-structure interaction (FSI) analyses have been useful in assessing MV biomechanics under healthy and diseased conditions, as well as in evaluating the functional effects of different surgical and transcatheter MV repair techniques (Wang et al., 2014; Caballero et al., 2018, 2019b, 2020; Sacks et al., 2019; Kong et al., 2020). While several computer studies have modeled the edge-to-edge and MitraClip procedures under degenerative or primary MR (Mansi et al., 2012; Zhong et al., 2014; Sturla et al., 2015; Morgan et al., 2017; Sturla et al., 2017; Prescott et al., 2019), to the best of our knowledge, no computer FSI study has to date evaluated the impact of MitraClip on left heart (LH) dynamics under functional MR. Previously, Lau et al. (2011) investigated the effect of the edge-to-edge surgical technique on an idealized MV model with a dilated static mitral annulus (MA) and healthy chordae structure with no leaflet tethering.

In this study, a previously validated patient-specific LH model with functional MR and heart failure (Caballero et al., 2019a) was used to: (1) simulate and evaluate MitraClip implantation with different clipping configurations and combined annuloplasty procedures, and (2) investigate the post-procedure LH dynamics throughout the cardiac cycle in order to quantify the acute changes in MR severity and assess the immediate biomechanical outcomes of the MitraClip procedure. Albeit a single patient case, we believe that this study offers a novel and detailed engineering analysis that could shed some light on the biomechanical impact of MitraClip on cardiac function in heart failure patients with significant MR. Further development and validation of such computer models could provide useful information toward proper patient selection and procedural optimization for treatment with transcatheter MV repair devices.

## Materials and Methods

### Patient-Specific LH Model With Functional MR

In this study, we employed a patient-specific LH model with functional MR, heart failure and reduced LV ejection fraction (LVEF) rigorously developed and validated in Caballero et al. (2019a). The use of de-identified patient clinical data for this study was approved by an Institutional Review Board. Briefly, cardiac multi-slice computed tomography (MSCT) images of a patient referred for transcatheter AV replacement (TAVR) were retrospectively collected from Hartford Hospital (Hartford, CT). Transthoracic echocardiographic (echo) examination revealed moderate-to-severe functional MR, with restricted posterior mitral leaflet (PML) motion and reduced leaflet coaptation, resulting in a posteriorly directed regurgitant jet. The LVEF was estimated to be 25%. The LV thickness was normal but the chamber was dilated with severe global hypokinesis with regional variation. The LA was dilated despite a normal antero-posterior diameter. Classical low-flow, low-gradient severe aortic stenosis (AS) was also found, with a bicuspid aortic valve (AV) with fused left and right coronary cusps.

**FIGURE 1:**
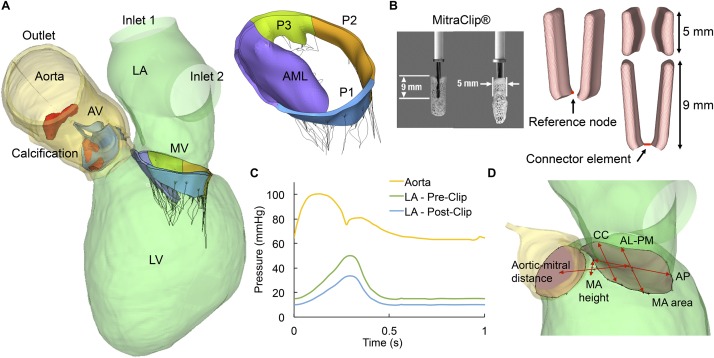
**(A)** Patient-specific LH model, **(B)** Real and simulated MitraClip devices, **(C)** Aortic and LA pressure waveforms, **(D)** MV geometrical parameters. AV, aortic valve; LV, left ventricle; LA, left atrium; MV, mitral valve; AML, anterior mitral leaflet; PML, posterior mitral leaflet is divided into lateral P1 scallop, central P2 scallop and medial P3 scallop.

The patient-specific LH model, as shown in Figure 1A, is composed of the ascending aorta, aortic root, AV, calcification, MV, and LV and left atrium (LA) endocardial walls. Additionally, the computer model comprises detailed mitral chordae structure and distribution, accurate leaflet geometry and thickness, dynamic MA and chordae origins, anisotropic hyperelastic material models, and human age- and gender-matched material properties. Chordae tethering forces due to papillary muscle (PM) displacement were accounted for accurate modeling of MV dynamics under functional MR, as previously presented in Pham et al. (2017). Further details on medical imaging segmentation, 3D model reconstruction, constitutive modeling, and model validation can be found in Supplementary Material.

### MitraClip and Annuloplasty FE Modeling

The clip device was modeled as two rectangular rigid plates mimicking the design and dimensions of the real MitraClip, as seen in Figure 1B. The length of each plate was 9 mm and the width was 5 mm. A reference node was defined at the middle bottom edge of each plate, allowing each arm to rotate with respect to its own reference node. A connector element connecting the two reference nodes was defined in order to impose mutual kinematic constraints. During the simulations, the relative motion of the two clip arms was constrained by the connector element to prevent relative displacement between them. Based on the location of the regurgitant gap, three MitraClip placement locations were simulated in this study: (i) lateral positioning, between the anterior mitral leaflet (AML) and P1 scallop, (ii) central positioning, between the AML and P2 scallop, and (iii) double positioning, by combining lateral and central clips.

Because multiple mechanistic etiologies of MR frequently coexist, there is a high clinical interest in combining or sequentially staging transcatheter approaches to eliminate MR, such as combined MitraClip with percutaneous annuloplasty (Rogers et al., 2018). Due to the lack of geometrical data for these novel transcatheter rings, the 3D profile of a well-established surgical annuloplasty ring (Carpentier-Edwards Classic) known to successfully treat functional MR by reducing the antero-posterior annular diameter was used in this study. Following the selection guidelines (Borghetti et al., 2000), a 38 ring size was chosen based on the surface area of the device and the AML. However, rather than modeling the implantation of the actual ring with its full cross-sectional geometry, ring implantation was simplified by displacing the nodes of the MA to the 3D shape of the ring, as previously presented in Kong et al. (2018). The 3D ring shape and dimensions were obtained from literature. Combined annuloplasty ring implantation was simulated for the central and double clip models. The MV repair procedures were modeled in three major FE steps:

#### Step 1: MV Closure

Simulation of MV closure under systolic pressure and chordae tethering has been described in detail in our previous studies (Pham et al., 2017; Kong et al., 2018). Briefly, since the heart failure patient had moderate-to-severe MR with PML tethering and dilated LV, pre-existing forces within the chordae at diastole were modeled in the first sub-step of the simulation. Thus, before running the FE simulation, a total of 8 posterior chordae were shortened by translating the chordae origins toward the MA plane along the original direction of the chordae. At the beginning of the step, those chordae origins were initially displaced to their original locations to generate the tethering tension. After those chordae origins reached their original locations on the PM tips, a rough contact with no separation behavior was enforced to connect the chordae with the PM tips (Pham et al., 2017). The reaction force on each node of the MA was output at the end of this first sub-step. Next, the dynamic motion of the MA and chordae origins from diastole to systole obtained from the MSCT images was applied as a nodal displacement boundary condition (Pham et al., 2017). The clinically measured trans-mitral pressure gradient of 114 mmHg was then applied to the ventricular surface of the leaflets to simulate MV closure. At the end of this step, the two MitraClip arms rotated so that the clip opened at 120 degrees.

#### Step 2: Clip Grasping

Clip grasping to the mitral leaflets was modeled at diastole when the valve just started to open. Initially, displacement boundary conditions on the MA and chordae were applied so that the mitral apparatus restored to a diastolic position and the leaflets opened due to the release of the pressure on the ventricular surface. Next, the MA nodal displacement boundary condition was replaced by the MA reaction forces obtained in Step 1 from the pretension simulation at diastole to account for the tension within the MA during diastole. Meanwhile, to grasp the leaflets, the two clip arms rotated back to a closed position until a gap of 4 mm was left between the upper end of the two arms (Magruder et al., 2016). At the end of the step, rough contact with no slippage and no separation behavior was initiated between the clip arms and the mitral leaflets. The arms remained mutually parallel but could rotate/tilt following the interaction with the leaflets.

#### Step 3: Annuloplasty Ring Implantation

To align the virtual ring with the MA plane, least-square planes were created for both the ring and the MA. Next, middle anterior and posterior portions of the ring were aligned with the middle anterior and posterior portions of the MA until the ring was positioned such that the anterior portion of the ring overlapped with the anterior MA, avoiding excessive displacement of the LV outflow tract (LVOT). Following ring alignment, a total of 18 node clusters uniformly distributed along the MA were identified as boundary nodes; each cluster contained 3 adjacent nodes. On the virtual ring, 18 uniformly distributed nodes were also identified. Suturing of the annuloplasty ring to the MA was simulated by imposing kinematic displacements on the 18 node clusters on the MA from their original locations to the locations of the 18 corresponding nodes identified on the virtual ring (Kong et al., 2018).

During all FE simulation steps that modeled the MitraClip and annuloplasty implantation procedures, nodes on the septal wall of the LV myocardium were fixed to prevent excessive cardiac motion. The rest of the LV was not constrained to allow deformation near the MA during the repair therapies. The resulting deformed LH geometries after MitraClip and annuloplasty procedures were extracted from the FE simulations and used to assess the post-procedure LH dynamics using FSI.

### FSI Modeling of Pre- and Post-procedure LH Dynamics

The FSI modeling framework used in this study has been previously developed, validated and implemented to evaluate the LH dynamics under a variety of physiologic, pathologic and repaired states (Mao et al., 2016a, 2017; Caballero et al., 2017, 2018, 2019a,b). Briefly, the FSI approach combines smoothed particle hydrodynamics (SPH) for the blood flow and non-linear FE analysis for the heart valves. As seen in Figure 1C, time-dependent pressure boundary conditions were applied at the two LA inlets (pulmonary veins) and at the aortic outlet of the pre- and post-procedure models. In functional MR, the regurgitant volume in the LA results in an elevated V-wave pressure during systole (Mokadam et al., 2011). After MitraClip, the LA pressure decreased by 33% (Kuwata et al., 2019; Turyan Medvedovsky et al., 2019). On the outlet, a physiologic aortic pressure waveform was employed. These waveforms were fitted to match this particular patient’s pressure values clinically measured (Caballero et al., 2019a).

Endorcardial LV and LA wall motion and chordae origins motion during the pre- and post-procedure FSI simulations were imposed as a time-dependent nodal displacement boundary condition based on the MSCT images (Caballero et al., 2017; Mao et al., 2017). This cardiac wall motion was kept the same for all pre- and post-procedure models, simulating immediate post-operative LH dynamics, without considering any possible cardiac remodeling mechanisms that occur over time after MV repair. SPH particles were uniformly distributed in the LH domain with a spatial resolution of 0.8 mm and given Newtonian blood properties with a density of σ = 1056 kg/m^3^ and a dynamic viscosity of μ=0.0035 Pa.s. SPH particle sensitivity (Mao et al., 2016b; Caballero et al., 2017) and FE mesh sensitivity (Wang and Sun, 2013) studies were previously performed. The patient’s heart rate was approximately 60 bpm, corresponding to a cardiac cycle of 1 s. Two cardiac cycles were conducted and the results from the second cycle were analyzed in this study. Abaqus/Explicit 6.17 (3DS, Dassault Systéms, Paris, France) was used for all FE and FSI simulations presented in this work.

### Data Analysis

#### MV Geometrical Parameters

Morphologic changes in the MV during MitraClip were evaluated in terms of the geometrical parameters shown (Figure 1D). The following measurements were obtained during systole: (a) antero-posterior (AP) distance, (b) anterolateral-posteromedial (AL-PM) distance, (c) MA spherecity index (ASI), defined as the ratio between AP and AL-PM distances, (d) inter-commissural (CC) distance, (e) MA height to inter-commissural width ratio (AHCWR), defined as the ratio between MA height and CC distance, (f) MA area, and (g) aortic-mitral distance, defined as the centroid distance between the mitral and aortic annuli.

#### Fluid Parameters

**FIGURE 2:**
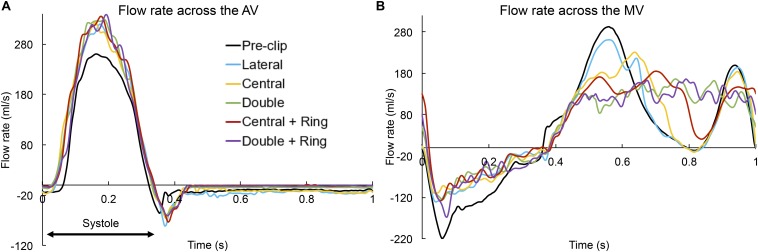
Flow rate (ml/s) across the **(A)** AV and **(B)** MV throughout the cardiac cycle.

The following hemodynamic parameters were quantified throughout the cardiac cycle: (a) stroke volume in the AV (*SV*_*AV*_) and MV (*SV*_*MV*_), obtained by integrating the positive aortic and mitral flow over time, respectively (Figure 2), (b) regurgitant volume in the AV (*RV*_*AV*_) and MV (*RV*_*MV*_), obtained by integrating the negative aortic and mitral flow over time, respectively (Figure 2). The regurgitant volume was defined as the sum of the valve closing and the leakage volumes, (c) regurgitant fraction, *RF*_*MV*_ = *RV*_*MV*_/*LVSV* where *LVSV* is the total *SV* of the LV (*SV*_*AV*_+ *RV*_*MV*_), (d) MR severity, graded using the *RF*_*MV*_ criterion (Zoghbi et al., 2003), (e) LVEF, (f) LV mean systolic pressure (LV-MSP), (g) LV end-diastolic pressure (LV-EDP), (h) peak systolic pressure gradient (PSPG), (i) mean systolic pressure gradient (MSPG), (j) AV peak velocity, (k) AV effective orifice area, $E\,O\,A_{A\,V}=\frac{M\,S\,F}{51.6\,\sqrt{\text{MSPG}}}$, where *MSF* is the root mean square systolic flow rate (Saikrishnan et al., 2014), (l) peak diastolic pressure gradient (PDPG), (m) mean diastolic pressure gradient (MDPG), (n) E and A wave velocities, (o) MV effective orifice area, $E\,O\,A_{M\,V}=\frac{M\,D\,F}{31\,\sqrt{\text{MDPG}}}$, where *MDF* is the root mean square diastolic flow rate (Chandran et al., 2012), (p) MR mean pressure gradient (MR-MPG), q) MR mean velocity, and (r) effective regurgitant orifice area, $E\,R\,O\,A=\frac{M\,R\,F}{31\,\sqrt{\text{MR}-\text{MPG}}}$, where *MRF* is the root mean square regurgitant flow rate.

#### Structural Parameters

Pre- and post-procedure MV biomechanics were evaluated by the average maximum principal stress (S_I_^*AVRG*^) in the MV leaflets during peak systole and diastole. To avoid the bias caused by local high stress concentration, only the 99-percentile values of the peak stress values were evaluated (Auricchio et al., 2011). Moreover, MA regions were not included in the average stress calculation in order to avoid boundary effects. AML and PML chordae forces (F_chordae_) were also reported. The force experienced by a particular chordae group was calculated as the sum of vectors representing the tension in each individual chorda attached to a specific leaflet. Finally, the anatomic MV orifice area (AMVOA) was quantified throughout the cardiac cycle. The least square plane of the MA was calculated at each time point of the cardiac cycle. The AMVOA was then measured as the projected 2D area of the mitral leaflets free edge on the MA least square plane. The anatomic regurgitant and opening orifice areas were obtained by selecting the minimum and maximum AMVOA values during systole and diastole, respectively.

## Results

### MitraClip Impact on MV Geometry

Table 1 summarizes the changes in MV geometry during MitraClip implantation. The AP distance and ASI markedly decreased (>20%) for the central and double clip models, with or without ring. Similarly, the MA area and aortic-mitral distance had a tendency to decrease for all clip models, but these changes were only important for the double + ring model when compared to the pre-clip state. Furthermore, the AL-PM and CC distances showed a trend to increase after MV repair, however, these changes were relatively small and comparable before and after the procedure. Generally, using a lateral clip led to the smallest changes in MV geometry, while using a double clip + ring led to the biggest anatomic changes.

**TABLE 1:** MV anatomical parameters pre- and post-clip.

	Pre-clip	Lateral	Central	Double	Central + Ring	Double + Ring
AP distance (mm)	34.37	31.90	27.37 (−20)	27.66 (−20)	25.28 (−26)	24.74 (−28)
AL-PM distance (mm)	39.83	41.96	43.28	43.51	45.71	46.80
ASI	0.86	0.76	0.63 (−27)	0.64 (−26)	0.55 (−36)	0.53 (−39)
CC distance (mm)	33.87	35.22	34.90	35.36	37.42	37.98
AHCWR	0.14	0.16	0.11	0.12	0.12	0.14
MA area (cm^2^)	11.40	10.37	10.04	9.58	9.40	9.14 (−20)
Aortic-mitral distance (mm)	28.32	25.06	24.43	23.71	23.38	22.78 (−20)

*Marked percentage variations (%) with respect to the pre-clip model are reported in parenthesis.*

### MitraClip Impact on Intraventricular Hemodynamics

Figure 2 shows the flow rate waveforms across the valves throughout the cardiac cycle for the pre- and post-clip models. The positive flow indicates the forward flow toward the aorta (Figure 2A) and the LV (Figure 2B) during systole and diastole, respectively. In contrast, the negative flow indicates the backward blood flow due to valve closure and regurgitation. Based on mass conservation and since blood is incompressible, *SV*_*AV*_ + *RV*_*MV*_ during systole = *SV_*M*__*V*_* + *RV_*A*__*V*_* during diastole (Mao et al., 2020). Additionally, Table 2 summarizes the main hemodynamic parameters quantified for the pre- and post-clip models from the FSI simulations.

**TABLE 2:** Pre- and post-clip LH hemodynamics.

	Pre-clip	Lateral	Central	Double	Central + Ring	Double + Ring
SV_AV_ (ml)	46.28	58.81	58.55	60.94	61.21	57.13
RV_AV_ (ml)	9.34	13.18	10.42	6.37	4.78	4.85
SV_MV_ (ml)	74.64	70.22	71.57	74.45	75.70	75.38
RV_MV_ (ml)	37.59	23.88	23.59	20.86	19.51	23.98
RF_MV_ (%)	44.82	28.88	28.72	25.50	24.17	29.56
MR severity (RF_MV_)	Moderate-to-severe	Moderate	Moderate	Moderate	Moderate	Moderate
LVEF (%)	28.55	28.15	27.96	27.84	27.47	27.61
LV-MSP (mmHg)	97.64	105.7	105.43	110.31	110.38	103.94
LV-EDP (mmHg)	15.96	9.67	8.39	0.03	0.92	0.01
PSPG (mmHg)	34.82	30.74	30.58	34.14	33.90	31.50
MSPG (mmHg)	23.97	20.97	20.7	21.68	21.34	20.84
AV peak velocity (m/s)	2.82	2.76	2.8	2.81	2.8	2.77
EOA_AV_ (cm^2^)	0.77	0.94	0.93	0.95	0.96	0.93
PDPG (mmHg)	4.32	8.91	9.05	10.4	9.17	10.78
MDPG (mmHg)	1.35	4.33	6.16	9.17	7.47	9.2
E wave (m/s)	0.79	0.96, 1.46	1.41, 1.29	1.15, 0.92, 1.44	1.43, 1.36	1.2, 0.74, 1.47
A wave (m/s)	0.54	0.71, 1.2	1.30, 1.15		1.31, 1.28	
EOA_MV_ (cm^2^)	4.1	2.19	1.76	1.32	1.53	1.32
MR-MPG (mmHg)	57.25	75.24	76.65	79.84	79.56	74.25
MR peak velocity (m/s)	5.42	4.81	4.83	4.9	4.89	4.82
EROA (cm^2^)	0.5	0.28	0.26	0.24	0.24	0.28

*E and A wave velocity values from left to right correspond to the double/triple orifices located from P1 to P3 scallops.*

**FIGURE 3:**
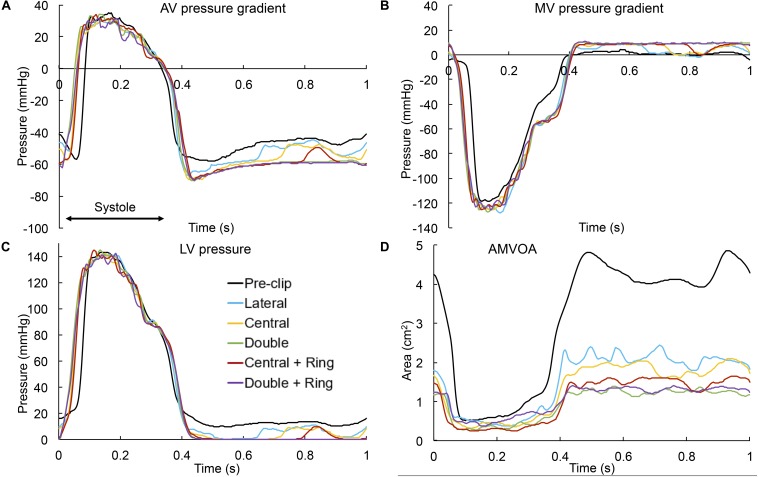
**(A)** AV pressure gradient, **(B)** MV pressure gradient, **(C)** LV pressure, and **(D)** AMVOA throughout the cardiac cycle.

Several important findings can be quantified during systole: First, MitraClip therapy led to an immediate hemodynamic improvement by decreasing the *RV*_*AV*_, and due to the coupled aortic-mitral valve dynamics and mass conservation, a concomitant increase in the forward *SV*_*AV*_ (Table 2). The greatest degree of MR improvement was for the central + ring model (46%). Moreover, all post-clip models can now be classified as moderate MR. Second, an increase in the LV-MSP of 8–13 mmHg was quantified immediately after MitraClip, reflecting the increase in the SV_*AV*_. Third, the MR-MPG increased after the procedure (Figure 3B) due to a better closure of the mitral leaflets, the decrease in the LA pressure, and the increase in the LV-MSP. The peak MR velocity and EROA consequently decreased after MitraClip. Fourth, improvement in the systolic hemodynamic profile after clip implantation was also associated with a decrease in the PSPG and MSPG (Figure 3A), and an increase in the EOA_*AV*_.

Similarly, important findings were quantified during the diastolic phase: First, narrowing of the MV orifice after MitraClip caused higher PDPG and MDPG (Figure 3B), as well as higher MV inflow velocities (Table 2). The MDPG increased from 1.35 mmHg pre-clip up to 9.2 mmHg when using a double clip + ring. Second, MR reduction promoted a decrease in the preload, manifested by a decrease in the LV-EDP (Figure 3C). The double clip models presented the greatest decrease in LV preload. Third, as seen in Figure 2B, the MV inflow curve profile changed after repair. Due to the decrease in the EOA_*MV*_ and the greater resistance to flow in the multiple-orifice MV, there was a decrease in the early E-wave dominant flow. This decrease was more dominant for the double clip models, which presented the lowest EOA_*MV*_.

**FIGURE 4:**
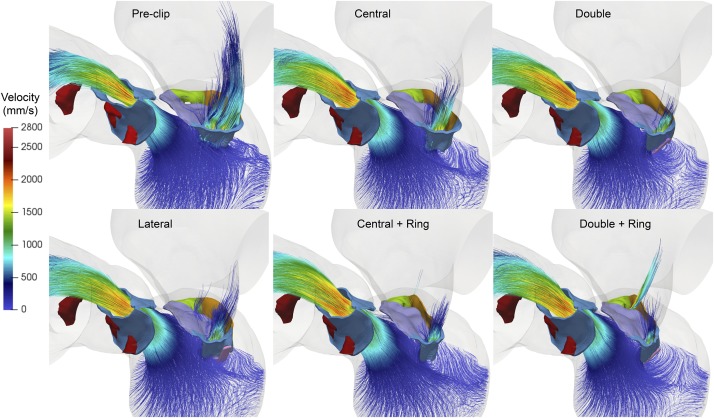
Pre- and post-clip velocity showing the regurgitant jet structures during peak systole.

Figure 4 shows the intraventricular velocity streamlines colored by velocity magnitude during peak systole. Due to restricted PML motion, the pre-clip model displayed a posteriorly directed regurgitant jet in the P1 region, which qualitatively and quantitatively matched the regurgitant jet measured clinically (Caballero et al., 2019a). The overall regurgitant jet direction was similar between pre- and post-clip states, with an eccentric “wall-hugging” jet that impinged the postero-lateral LA wall. The strength and velocity of the jet, however, decreased following MitraClip (Table 2). Moreover, when a double clip + ring was implanted, a second small regurgitant jet structure was visible in the postero-medial MA region, which supports the finding of the highest *RV*_*MV*_ between all post-clip models (Table 2).

**FIGURE 5:**
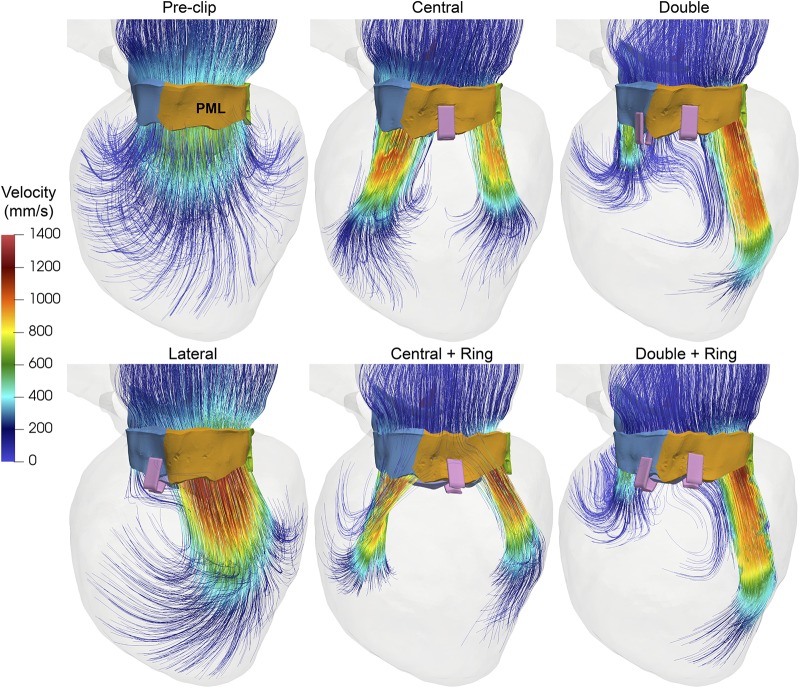
Pre- and post-clip velocity showing the intraventricular blood flow during peak diastole.

Finally, Figure 5 shows the velocity streamlines during peak diastole. Marked differences in the inflow flow structure and magnitude were observed pre- and post-clip, as well as between the different clip/ring configurations. Clip implantation significantly altered the intraventricular hemodynamics by creating a multiple-jet flow due to the double-orifice MV for the lateral and central clip models, and the triple-orifice MV for the double clip models. Moreover, the post-clip jets were not oriented toward the apex, but toward the LV wall where they impinged, leading to higher near-wall velocities than the pre-clip state, especially for the clip models with a ring. As shown in Table 2, due to the reduced EOA_*MV*_, the inflow jets had much higher velocities than the central jet observed before clip implantation.

### MitraClip Impact on Tissue Mechanics

**FIGURE 6:**
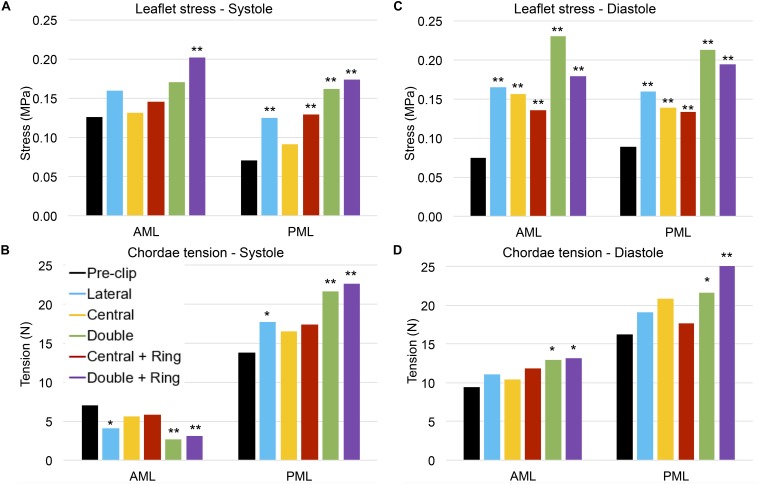
**(A)** Average MV leaflet stress during peak systole, **(B)** Chordae tension during peak systole, **(C)** Average MV leaflet stress during peak diastole, and **(D)** Chordae tension during peak diastole. ** highlight a 50% increase/decrease with respect to the pre-clip model, and * highlight a 30% increase/decrease with respect to the pre-clip model.

**FIGURE 7:**
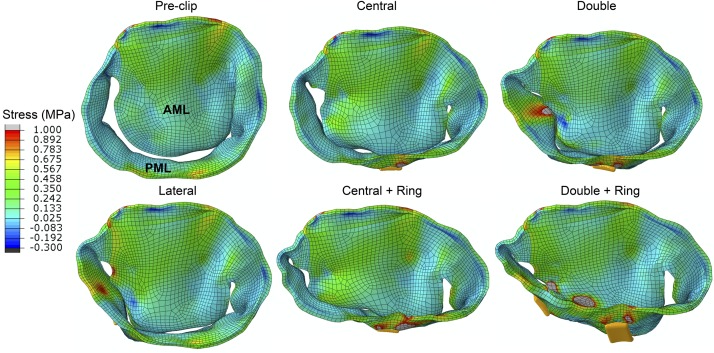
Stress distribution on the MV leaflets during peak systole.

Table 3 and Figure 6A present the average stress in the mitral leaflets during peak systole. From Figure 6A it can be seen that both AML and PML were subjected to a higher systolic stress after the procedure. This increase in leaflet stress was significant (>50%) in the PML for all post-clip models except when using a central clip. Additionally, Figure 7 shows the stress distribution across the MV leaflets at peak systole. Before clip implantation, peak stresses in the AML were located at the right and left trigones, while peak stresses for the PML were distributed along the MA region and close to the basal chordae insertion regions. Following clip attachment, areas of high stress concentration were relocated near the clip arms for all MitraClip configurations, and extended above the leaflet portion grasped by the clip arms toward the annular region. Repaired-induced leaflet stresses in areas remote from the devices were not significant.

**TABLE 3:** Pre- and post-clip MV biomechanics during peak systole.

	Pre-clip	Lateral	Central	Double	Central + Ring	Double + Ring
**S_I_^AVRG^ (MPa)**						
AML	0.126	0.159 (26)	0.131 (5)	0.170 (35)	0.145 (16)	0.202 (61)
PML	0.071	0.125 (76)	0.091 (28)	0.162 (128)	0.129 (82)	0.174 (145)
**F_chordae_ (N)**						
AML	7.04	4.07(−42)	5.59(−21)	2.66(−62)	5.88(−16)	3.16(−55)
PML	13.79	17.71 (28)	16.50 (20)	21.62 (57)	17.36 (26)	22.62 (64)
AMOVA - Regurgitant orifice area (cm^2^)	0.51	0.37(−29)	0.32(−38)	0.27(−47)	0.25(−52)	0.37(−28)

*Percentage variations (%) with respect to the pre-clip model are reported in parenthesis.*

Figure 6B presents the chordae tension during peak systole. Since this patient had a postero-lateral regurgitant gap with PML tethering, it can be seen that the pre- and post-clip models experienced a significantly higher total PML chordae tension when compared to AML chordae tension. After MitraClip, the chordae attached to the AML were subjected to a lower tension compared to the pre-clip model, while PML chordae were under a higher tension. These differences were significant (>50%) when two clips were implanted, with or without ring.

**FIGURE 8:**
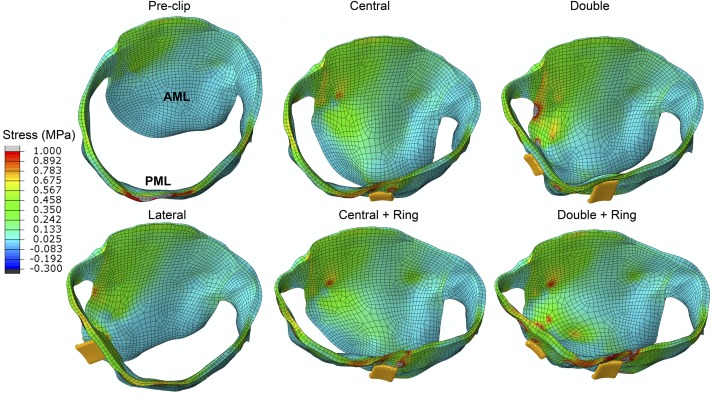
Stress distribution on the MV leaflets during peak diastole.

Regarding the diastolic phase, Table 4 and Figure 6C show a significant increase (>50%) in AML and PML average stress for all post-clip models. Moreover, the central clip + ring model gave the lowest increase in leaflet stress, while the double clip model gave the highest increase. As seen in Figure 8, similar stress distribution patterns were observed in the MV leaflets for all post-clip models during diastole. In the AML, peak stresses relocated near the clip arms and along strut and marginal chordae insertion regions. In the PML, peak stresses extended from the free edge at the level of the clip arms toward the P2 annular region, close to the insertion of the basal chordae.

**TABLE 4:** Pre- and post-clip MV biomechanics during peak diastole.

	Pre-clip	Lateral	Central	Double	Central + Ring	Double + Ring
**S_I_^AVRG^ (MPa)**						
AML	0.074	0.165 (122)	0.156 (110)	0.230 (210)	0.135 (82)	0.179 (142)
PML	0.088	0.159 (80)	0.139 (57)	0.213 (141)	0.133 (50)	0.195 (120)
**F_chordae_ (N)**						
AML	9.43	11.04 (17)	10.42 (11)	12.99 (38)	11.85 (26)	13.17 (40)
PML	16.25	19.06 (17)	20.92 (29)	21.65 (33)	17.63 (8)	27.81 (71)
AMVOA – Opening orifice area (cm^2^)	4.85	2.46(−49)	2.11(−56)	1.4(−71)	1.66(−66)	1.42(−71)

*Percentage variations (%) with respect to the pre-clip model are reported in parenthesis.*

In regard to diastolic chordae forces, Figure 6D shows that although AML and PML chordae tension had a tendency to increase after the procedure, these changes we only important (>30%) for the double clip models. When comparing systolic and diastolic chordae forces (Figures 6B,D) between each pre- and post-clip model, we observed a higher chordae tension during diastole, which could be attributed to the diastolic restriction of the mitral leaflets caused by PM relocation and LV dilatation.

Finally, Figure 3D shows the AMVOA throughout the cardiac cycle. During systole, the AMVOA markedly decreased (>28%) from 0.51 cm^2^ pre-clip up to 0.25 cm^2^ after MV repair, with the highest reduction (52%) obtained for the central clip + ring model (Table 3). During diastole, the AMVOA decreased significantly (>50%) for all post-clip models, with the highest reduction (71%) quantified for the double clip models (Table 4). Furthermore, as seen in Figure 3D, the pre-clip double-peaked AMVOA curve waveform changed to a flat curve post-clip, similarly as the MV inflow curves seen Figure 2B.

## Discussion

The results of this first-in-human experience with patient-specific computer simulations of MitraClip under functional MR can be summarized as follows: First, MitraClip induced acute morphologic changes in the mitral apparatus. Clip implantation resulted in a marked decrease of the AP distance by up to 28%, together with a small increase of the AL-PM. Consequently, there was a significant reduction of the ASI by up to 39%, together with a non-significant decrease in the AHCWR and MA area, suggesting that MitraClip leads to a more elliptical and planar MA. Second, the immediate reduction in the *RV*_*MV*_ achieved by MitraClip resulted in an acute increase in the forward flow (>23%), a decrease in preload, and overall improved hemodynamic profile. For this patient case, while implanting a central clip + ring resulted in the highest MR reduction (46%), this configuration also led to mitral stenosis (MS) by increasing the MDPG to 7.47 mmHg. Third, the MV leaflets were subjected to a higher loading state throughout the cardiac cycle after the procedure, with a significant increase in the diastolic leaflet average stress by up to 210 and 145% for the AML and PML, respectively. All post-clip models resulted in a concentrated high stress pattern at the region of clip grasp. Fourth, MitraClip was accompanied by the reduction of the anatomic opening area by up to 71% for the double clip models, while the anatomic regurgitant area decreased by up to 52% for the central clip + ring model, resulting in improved but incomplete MV coaptation. Finally, MitraClip altered the temporal course of the MV inflow and AMVOA diastolic waveforms from double-peaked to more flattened curves.

### Effects on MV Geometry

The acute effects of MitraClip on MV geometry under functional MR have been investigated in a few clinical studies using echo (Schmidt et al., 2013; Schueler et al., 2014; Noack et al., 2019b). Similar to these investigations, we found a marked reduction of the AP distance together with a relatively small increase of the AL-PM distance, especially when a ring was added (Table 1). These changes were accompanied by comparable changes in the CC distance pre- and post-clip (Donmez et al., 2019), and by a non-significant reduction in the MA area, as found in previous studies (Mantegazza et al., 2018; Noack et al., 2019b). Some studies, however, quantified a significant decrease of the MA area after MitraClip (Schmidt et al., 2013; Schueler et al., 2014). Nonetheless, this decrease in MA area appears to be secondary to reduced AP distance after the procedure. Our results suggest that reshaping of the MA with a significant reduction of the AP distance contributes to a reduction of MR after MitraClip, which might be an indicator of clinical benefit (Schueler et al., 2014; Patzelt et al., 2017; Schueler et al., 2017). As a whole, it appears that MitraClip limits MV further dilation by exerting traction on the MA (Mantegazza et al., 2018); mainly in the antero-posterior direction. The small changes in the CC distance can be explained by the presence of the aortic-mitral continuity and the fibrous trigones, which is a region less prone to large deformations than the posterior MA, which is mainly muscular and more susceptible to dilation (McCarthy et al., 2010).

The decrease of the AP distance together with the increase in the AL-PM distance resulted in a significant reduction of the ASI in the central and double clip models, which confirms the reshaping of the MA from a rounder to a more oval shape. The reduction in the MA sphericity has been quantified in patients with functional MR undergoing surgical MV repair with annuloplasty ring (Mahmood et al., 2010). AHCWR and aortic-mitral distance also had a tendency to decrease after MitraClip, but these changes were comparable pre- and post-clip. The AHCWR is a measure of the MA non-planarity and describes its 3D shape. Our results suggest a flatter MA post-clip, which can be related with the increased LV pressure and the more elliptical MA (Jimenez et al., 2006; Warraich et al., 2012). Overall, these geometric findings might be of interest for future biomechanical studies to determine independent anatomic features that can be correlated to sustained procedural success.

### Effects on MV Tissue Mechanics

MV leaflet stress increased throughout the cardiac cycle after MitraClip, especially in the vicinity of clip insertion (Zhang et al., 2019). More importantly, this increase was significant (50–210%) in all post-clip models during diastole (Table 4 and Figure 6C). These results are congruent with the findings from Lau et al. (2011) that showed that during diastole the MV leaflet stress can be up to 200% higher after MitraClip when treating MA dilatation. This important local increase in leaflet peak stress could be a key factor in triggering mitral leaflet remodeling after MitraClip. *In vitro* studies have shown that increased leaflet stress can alter proteoglycan and collagen synthesis, resulting in leaflet thickening with an increased compliance (Quick et al., 1997; Kunzelman et al., 1998). Moreover, under *in vivo* conditions the MitraClip has been reported to gradually be encapsulated by connective tissue, have thicker fibrous capsules, and incite a fibrous reaction that results in the formation of a tissue bridge within the clip arms (Ladich et al., 2011).

Although AML and PML had comparable stress values for each post-clip model during diastole (Table 4 and Figure 6C), the leaflets were subjected to abnormal stresses up to 52% higher with respect to the systolic phase (Figure 6A). For the clip models with ring, however, the leaflets were subjected to comparable stress values during systole and diastole. Thus, the use of an annuloplasty ring in combination with MitraClip appears to induce a more homogeneous stress state in the leaflets throughout the cardiac cycle. This key finding can have an important impact upon transcatheter MV repair techniques and their durability, whereby MitraClip is the only repair procedure currently performed.

### Effects on Anatomic MV Orifice Area

We report, to our knowledge, the first set of time-dependent AMVOA measurements pre- and post-clip under different clipping configurations and combined annuloplasty procedures (Figure 3D). The computer-based approach implemented herein allowed us to directly quantify the true MV orifice area at the level of the leaflet free edge with high reproducibility and accuracy. Clinically, this parameter could be assumed to represent an invasive measurement using catheterization or the “ground truth.” Nevertheless, for better comparability with clinical data, we also quantified the EOA_*MV*_ and EROA by using the mathematical formulation that relates the pressure drop across the valve and the flow rate (Chandran et al., 2012; Saikrishnan et al., 2014). We measured a reduction in the EOA_*MV*_ and EROA after MitraClip by up to 68 and 52%, respectively (Table 2). Congruent with our results, a recent 3D TEE study by Noack et al. (2019b) found that the EOA_*MV*_ and EROA decreased by up to 65 and 67%, respectively. Previous works have shown a decrease in the EOA_*MV*_ of ∼53% after the procedure (Altiok et al., 2012; Biaggi et al., 2013). In these studies, the EOA_*MV*_ was calculated with 3D planimetry in the proximity of the commissures, which can lead to an overestimation of the post-clip EOA_*MV*_. This could help to explain the larger decrease in EOA_*MV*_ quantified in our study.

Clinical assessment of the continuous MV orifice area during MitraClip is technical challenging due to the 3D dynamic MV orifice, the limited capacity for echo measurement of areas below 0.5 cm^2^, and the different TEE and Doppler-derived measurement techniques (Biaggi et al., 2013). When comparing our AMVOA values (Tables 3, 4) with the EOA_*MV*_ and EROA values (Table 2), we observed the same trend toward a decrease of the MV opening and regurgitant orifice areas after MitraClip. However, we also quantified that overall, the echo-based method (EOA_*MV*_ and EROA) underestimates the true MV orifice (AMVOA) (Biaggi et al., 2013), especially the regurgitant orifice area, with an error up to 24% for the double clip + ring model. Interestingly, out study also showed that MitraClip therapy altered the temporal MV leaflet kinematics, especially during diastole. A seen in Figure 3D, before MitraClip, a twin-peaked AMVOA curve was quantified, which can be explained by early MV inflow (E wave) followed by atrial contraction (A wave). After clip implantation, the double-peaked AMVOA curve changed to a more flattened curve as more clips were implanted. Although some clinical studies have detected this diastolic flattening of the AMVOA curve, it was suggested this was caused by the prevalence of atrial fibrillation in the patients studied (Noack et al., 2019a). But unlike previous reports, we prescribed the same LA wall motion pre- and post-clip in our virtual patient-specific model. Thus, we hypothesize these diastolic changes in the profile of the AMVOA and MV inflow curves post-clip are related to the MS caused by the narrower and multiple-orifice MV.

### Effects on Blood Flow Dynamics

#### Impact on Residual MR

The most important clinical hemodynamic parameters that determine MitraClip procedural success are residual MR and transmitral pressure gradient (MDPG). Even moderate MR after the procedure has been associated with increased mortality (Buzzatti et al., 2016), particularly in patients with impaired LV function and heart failure (Cheng et al., 2017). Indeed, residual MR is presented as one of the main drivers for worse patient outcome in the MITRA−FR trial, compared with the COAPT trial, with 50% residual MR ≥ 2 in MITRA−FR, and 31% residual MR ≥ 2 in COAPT after 1 year (Obadia et al., 2018; Stone et al., 2018). After MitraClip, all our post-procedure models decreased MR severity to moderate (Table 2). This finding of moderate MR would be considered a suboptimal clinical outcome (Paranskaya et al., 2013), leading to the consideration to deploy additional clips. As a general guideline, additional clips should not be placed if the patient has a mean MDPG ≥ 4 mmHg (Singh et al., 2015), which was the case for all our post-clip models. Regardless of whether 1 or more clips are deployed, current clinical MR assessment relies heavily on echo evaluation by integrating multiple parameters (Stone et al., 2015; Zoghbi et al., 2017; Mao et al., 2020), some of which are not validated in the specific MitraClip clinical scenario, are limited by operator dependence, or are difficult to obtain with TEE imaging (Krieger et al., 2016; Palmiero et al., 2017; Corrigan et al., 2018; Dietl et al., 2018).

Despite the reassuring data on efficacy and long-term durability of MitraClip, the proportion of patients with residual MR after therapy is not negligible. In light of the detrimental prognostic impact of MR, there is a high clinical interest in combining or sequentially staging transcatheter approaches to eliminate MR. Combined percutaneous therapies can be performed together at the time of initial treatment, or they can be staged for the treatment of persistent or recurrent MR (Rogers et al., 2018). For example, Latib et al. (2016) reported initially using 2 clips in a patient with functional MR, followed by staged CardioBand (Edwards Lifesciences, Irvine, CA, United States) transcatheter annuloplasty for persistent MR. In our study, the greatest degree of MR improvement was found for the central clip model followed by annuloplasty. From surgical experience, we know that annuloplasty as a part of MV repair stabilizes the MA and improves long-term results. In the near future and with the rapidly expanding arena of catheter-based technologies for MV repair, the combined application of multiple devices will increase the therapeutic arsenal, optimize results, and expand the pool of treatable patients to include those with multiple mechanistic etiologies of MR.

#### Impact on MV Diastolic Pressure Gradient

The benefit from MR reduction can be counterbalanced by the formation of MS, which is associated with the creation of a multiple-orifice MV (Herrmann et al., 2006; Boerlage-van Dijk et al., 2014). The abrupt change of a hemodynamic status from an elevated preload caused by MR, to an elevated after load caused by MS and the decrease of the low-impedance regurgitant flow into the LA can have a major impact on procedural outcomes. A recent study by Neuss and colleagues (Neuss et al., 2017) found that MS after MitraClip has a negative impact on long-term clinical outcomes. A cutoff value was found at 5 mmHg for invasively and 4.4 mmHg for echo measured MDPG. Based on the invasively threshold value, implantation of central and double clips, with or without ring, caused MS in our virtual patient models. In clinical practice, the pressure drop across the MV during MitraClip is usually calculated using the simplified Bernoulli equation and the maximum velocity measured by TEE or Doppler-derived echo (Bach, 2010; Zoghbi et al., 2009). Echo measurements are operator dependent and strongly influenced by LV function, LA compliance, and loading conditions (Kang et al., 2013). In addition, some Doppler methods have not been adequately validated in a multiple-orifice MV (Kar and Sharma, 2015). Considering these limitations, evaluation of directly measured hemodynamic parameters pre- and post-clip should be included to support decision making (Mao et al., 2020). In our study, all hemodynamic variables, including the MDPG and residual MR were quantified accurately and objectively using the pressure and velocity fields obtained from the simulation algorithms.

#### Impact on Intraventricular Hemodynamics

This study detected potentially harmful changes in the LV blood flow dynamics. While lateral clipping demonstrated a diastolic flow field closest to that of the pre-clip state, multiple diastolic jets were formed when central and double clips were implanted. The peak velocities across the MV were strongly affected by this multiple−orifice configuration (Table 2). From Figure 5 it is clear that the jets impinged the LV wall with a deeper penetration as they were deviated laterally due to the reduction in the MV orifice area. Moreover, the inflow velocities, trans-mitral pressure gradients, and jet deflection angle further increased when a ring was added. This deflection can compromise the fluid mechanics such as rapid dissipation of the large anterior vortex (Hu et al., 2010; Jeyhani et al., 2018), and a higher energy loss (Hu et al., 2010; Du et al., 2014). Although previous computer and *in vitro* studies have provided initial insights on the LV flow after MitraClip (Redaelli et al., 2001; Shi and He, 2009), simplified models that considered idealized LV and MV geometries were used. An improved understanding of human host-MitraClip hemodynamics using patient-specific FSI models may help to optimize device placement. We are currently working on performing a quantitative analysis of the intraventricular energetic parameters associated with maladaptive changes and LV reshaping in these FSI models (Cimino et al., 2017; Filomena et al., 2019).

### Clinical Implications

In daily MitraClip routine, the interventional team has to face several difficult situations from a decision-making standpoint: What should be done in case of residual MR?, what should be done in case of MR reduction, but a significant increase in the MDPG?, what is an acceptable compromise between these two parameters when implanting multiple clips? The answers to these questions are not always clear, suggesting that procedural assessment should be performed in a more integrated way. For example, real-time monitoring of LA pressure during MitraClip has been described as a helpful tool to predict clinical outcomes (Eleid et al., 2015; Horstkotte et al., 2016). Kuwata et al. (2019) demonstrated that an increased LA mean pressure was predictive of worse clinical outcomes at short-term follow-up, independent from echo findings. As intra-procedural decisions have a strong impact on short- and long-term outcomes and prognosis, the clinical message from these clinical studies is extremely relevant for the interventional community: evaluation of MitraClip should shift from a solely echo-based color Doppler assessment to a more hemodynamic-based approach.

To the best of our knowledge, this is the first patient-specific computer-based engineering study to quantify the coupled LH hemodynamics and tissue mechanics pre- and post-clip under different MitraClip/annuloplasty configurations throughout the cardiac cycle in a virtual human beating-heart. FSI modeling tools, as used in this study, are required to directly quantify the *RV*_*MV*_, accurately simulate full dynamic AV-MV dynamics (Lau et al., 2010; Mao et al., 2016b), and shift the conventional MitraClip paradigm from a solely anatomic assessment, toward a more functional and physiologic approach based on objective biomechanical data. Much needed clinically relevant flow indicators can be obtained by applying the computer modeling framework herein proposed for the personalized assessment of MitraClip therapy, as well as to gain insight into the different clinical scenarios and implantation criteria currently critical for this procedure. Moreover, this modeling methodology could be easily applied to other structural MV interventions and newer device designs.

### Limitations

The present study has several limitations. The main one is its small sample size. This work only used one previously validated patient-specific LH model with heart failure and functional MR (Caballero et al., 2019a), therefore no general statements can be made. A large cohort of patient-specific LH models under different MR conditions and MitraClip configurations would be necessary to draw conclusions with confidence. Second, simulations results were not validated against post-procedural clinical data, since the patient studied herein did not undergo a real MitraClip treatment. Nevertheless, this study allowed for a systematic investigation of various biomechanical scenarios resulting from combined MitraClip/annuloplasty procedures. Such well-controlled side-by-side comparisons under the same patient and loading conditions are challenging to obtain in a clinical setting. Third, annuloplasty procedures were simplified as nodal displacement boundary conditions on the MA, and the ring geometry was based on a surgical device. Fourth, some of the pressure and flow fluctuations seen in Figures 2, 3 after MitraClip implantation are thought to be caused by small numerical artifacts in the FSI simulations. Due to the use of prescribed cardiac wall motion and the assumption of incompressible fluid, a small compression in volume for a closed system can cause large changes in pressure and velocity. These fluctuations, however, damped out rapidly due to the viscous effect of the fluid. This numerical artifact could be resolved by including the shock-absorbing effect of the myocardium, and modeling its interaction with the blood considering active contraction. This is the subject of a study that we are currently undertaking. Finally, although cardiac tissue properties were age- and gender-matched, they were not patient-specific. Estimation of *in vivo* material parameters inversely from medical images would be ideal to produce more accurate predictive results (Liu et al., 2017).

## Conclusion

Percutaneous MV repair using MitraClip has been established as an option for heart failure patients with functional MR who failed medical therapy. Feasibility and safety of MitraClip has been largely described in a variety of clinical trials and case-reports. Results concerning efficacy and durability, however, are not entirely satisfactory. Additionally, results tend to be less impressive immediately after the procedure compared to surgical MV repair. In this era of booming technology, the collaboration between industry and academia is of utmost importance in order to bring further advancements in the field of percutaneous treatment of MV disease. The objective of this study was to evaluate the acute LH hemodynamic, structural and morphologic changes after MitraClip/annuloplasty therapies. Although this patient-specific computer study provided further evidence to support that MitraClip is a viable approach to treat functional MR by reducing regurgitation severity and improving LV systolic function, clip implantation also imposed a non-physiologic configuration and loading on the LV-valve complex, especially during diastole. Comprehensive personalized engineering analyses, as performed in this study, can be a powerful and versatile tool that can pinpoint specific biomechanical implications and potentially play an important role in elucidating the optimal setting and efficacy of percutaneous MV repair procedures.

## Data Availability Statement

All datasets generated for this study are included in the article/Supplementary Material.

## Ethics Statement

The studies involving human participants were reviewed and approved by Institutional Review Board of the Hartford Hospital. Written informed consent for participation was not required for this study in accordance with the national legislation and the institutional requirements.

## Author Contributions

AC and WS contributed to the conceptualization and project administration. AC contributed to the formal analysis, the methodology, the visualization, and the writing of the original draft. AC, WM, and WS contributed to the investigation. RM, RH, and WS contributed to the resources. WS contributed to the supervision. AC, WM, RM, RH, and WS contributed to the review and editing of the manuscript.

## Conflict of Interest

WS was a co-founder and serves as the Chief Scientific Advisor of Dura Biotech. He receives compensation and owns equity in the company. RH was a speaker for Abbott Vascular, Boston Scientific, Edwards Lifesciences, Philips Healthcare, and on the advisory board/consultant for Edwards Lifesciences, Gore & Associates, Medtronic, and Navigate. The remaining authors declare that the research was conducted in the absence of any commercial or financial relationships that could be construed as a potential conflict of interest.

## References

[B1] Ailawadi G. Lim D. S. Mack M. J. Trento A. Kar S. Grayburn P. A. (2019). One-year outcomes after MitraClip for functional mitral regurgitation. *Circulation* 139 37–47. 10.1161/CIRCULATIONAHA.119.040735 30586701

[B2] Altiok E. Hamada S. Brehmer K. Kuhr K. Reith S. Becker M. (2012). Analysis of procedural effects of percutaneous edge-to-edge mitral valve repair by 2D and 3D echocardiography. *Circ. Cardiovasc. Imaging* 5 748–755. 10.1161/CIRCIMAGING.112.974691 23001897

[B3] Auricchio F. Conti M. De Beule M. De Santis G. Verhegghe B. (2011). Carotid artery stenting simulation: from patient-specific images to finite element analysis. *Med. Eng. Phys.* 33 281–289. 10.1016/j.medengphy.2010.10.011 21067964

[B4] Bach D. S. (2010). Echo/Doppler evaluation of hemodynamics after aortic valve replacement: principles of interrogation and evaluation of high gradients. *JACC Cardiovasc. Imaging* 3 296–304. 10.1016/j.jcmg.2009.11.009 20223428

[B5] Biaggi P. Felix C. Gruner C. Herzog B. A. Hohlfeld S. Gaemperli O. (2013). Assessment of Mitral valve area during Percutaneous mitral valve repair using the mitraclip system comparison of different echocardiographic methods. *Circ. Cardiovasc. Imaging* 6 1032–1040. 10.1161/Circimaging.113.000620 24134955

[B6] Boerlage-van Dijk K. Van Riel A. C. de Bruin-Bon R. H. Wiegerinck E. M. Koch K. T. Vis M. M. (2014). Mitral inflow patterns after MitraClip implantation at rest and during exercise. *J. Am. Soc. Echocardiogr.* 27 24–31. 10.1016/j.echo.2013.09.007 24161483

[B7] Borghetti V. Campana M. Scotti C. Domenighini D. Totaro P. Coletti G. (2000). Biological versus prosthetic ring in mitral-valve repair: enhancement of mitral annulus dynamics and left-ventricular function with pericardial annuloplasty at long term. *Eur. J. Cardiothorac. Surg.* 17 431–439. 10.1016/s1010-7940(00)00344-4 10773567

[B8] Buzzatti N. De Bonis M. Denti P. Barili F. Schiavi D. Di Giannuario G. (2016). What is a “good” result after transcatheter mitral repair? Impact of 2+ residual mitral regurgitation. *J. Thorac. Cardiovasc. Surg.* 151 88–96. 10.1016/j.jtcvs.2015.09.099 26545970

[B9] Caballero A. Mao W. Liang L. Oshinski J. Primiano C. McKay R. (2017). Modeling left ventricular blood flow using smoothed particle hydrodynamics. *Cardiovasc Eng Techn.* 8 465–479. 10.1007/s13239-017-0324-z 28744784 PMC5709227

[B10] Caballero A. Mao W. McKay R. Primiano C. Hashim S. Sun W. (2018). New insights into mitral heart valve prolapse after chordae rupture through fluid–structure interaction computational modeling. *Sci. Rep.* 8:17306. 10.1038/s41598-019-44072-y 30470812 PMC6251907

[B11] Caballero A. Mao W. McKay R. Sun W. (2019a). The impact of balloon-expandable transcatheter aortic valve replacement on concomitant mitral regurgitation: a comprehensive computational analysis. *J. R. Soc. Interf.* 16:20190355. 10.1098/rsif.2019.0355 31409236 PMC6731489

[B12] Caballero A. Mao W. McKay R. Sun W. (2019b). Transapical mitral valve repair with neochordae implantation: FSI analysis of neochordae number and complexity of leaflet prolapse. *Int. J. Numerical Methods Biomed. Eng.* 2019:e3297. 10.1002/cnm.3297 31833663

[B13] Caballero A. Mao W. McKay R. Sun W. (2020). The impact of self-expandable Transcatheter Aortic valve replacement on concomitant functional mitral regurgitation: a comprehensive engineering analysis^∗^. *Struct. Heart* 1–13. 10.1080/24748706.2020.1740365 PMC7958485 33728393

[B14] Chandran K. B. Rittgers S. E. Yoganathan A. P. (2012). *Biofluid Mechanics: The Human Circulation.* Boca Raton, FL: CRC press.

[B15] Cheng R. Dawkins S. Tat E. Makar M. Hussaini A. Makkar R. R. (2017). Relation of residual mitral regurgitation despite elevated mitral gradients to risk of heart failure hospitalization after MitraClip repair. *Am. J. Cardiol.* 120 1595–1600. 10.1016/j.amjcard.2017.07.027 29025679

[B16] Cimino S. Palombizio D. Cicogna F. Cantisani D. Reali M. Filomena D. (2017). Significant increase of flow kinetic energy in “nonresponders” patients to cardiac resynchronization therapy. *Echocardiography* 34 709–715. 10.1111/echo.13518 28332315

[B17] Corrigan F. E. III Chen J. H. Maini A. Lisko J. C. Alvarez L. Kamioka N. (2018). Pulmonary venous waveforms predict rehospitalization and mortality after percutaneous mitral valve repair. *JACC* 12 1905–1913. 10.1016/j.jcmg.2018.07.014 30219407

[B18] de Jaegere P. Rocatello G. Prendergast B. D. de Backer O. Van Mieghem N. M. Rajani R. (2019). Patient-specific computer simulation for transcatheter cardiac interventions: what a clinician needs to know. *Heart* 105(Suppl. 2), s21–s27. 10.1136/heartjnl-2018-313514 30846521

[B19] Dietl A. Prieschenk C. Eckert F. Birner C. Luchner A. Maier L. S. (2018). 3D vena contracta area after MitraClip© procedure: precise quantification of residual mitral regurgitation and identification of prognostic information. *Cardiovasc. Ultrasound* 16:1. 10.1186/s12947-017-0120-9 29310672 PMC5759791

[B20] Donmez E. Salcedo E. E. Quaife R. A. Burke J. M. Gill E. A. Carroll J. D. (2019). The acute effects of edge-to-edge percutaneous mitral valve repair on the shape and size of the mitral annulus and its relation to mitral regurgitation. *Echocardiography* 36 732–741. 10.1111/echo.14284 30801804

[B21] Du D. Jiang S. Wang Z. Hu Y. He Z. (2014). Effects of suture position on left ventricular fluid mechanics under mitral valve edge-to-edge repair. *Biomed. Mater. Eng.* 24 155–161. 10.3233/BME-130795 24211894

[B22] Eleid M. F. Sanon S. Reeder G. S. Suri R. M. Rihal C. S. (2015). Continuous left atrial pressure monitoring during MitraClip: assessing the immediate hemodynamic response. *JACC Cardiovasc. Interv.* 8 e117–e119. 10.1016/j.jcin.2015.02.010 26003021

[B23] Filomena D. Cimino S. Maestrini V. Cantisani D. Petronilli V. Mancone M. (2019). Changes in intraventricular flow patterns after MitraClip implant in patients with functional severe mitral regurgitation. *J. Am. Soc. Echocardiogr.* 32 1250–3e1. 10.1016/j.echo.2019.05.022 31311702

[B24] Goldberg S. L. (2019). Reflections on percutaneous therapies for secondary mitral regurgitation. *Cardiovasc. Revasc. Med.* 20 528–529. 10.1016/j.carrev.2019.02.027 30905660

[B25] Grayburn P. A. Sannino A. Packer M. (2019). Proportionate and disproportionate functional mitral regurgitation a new conceptual framework that reconciles the results of the MITRA-FR and COAPT trials. *JACC Cardiovasc. Imaging* 12 353–362. 10.1016/j.jcmg.2018.11.006 30553663

[B26] Herrmann H. C. Rohatgi S. Wasserman H. S. Block P. Gray W. Hamilton A. (2006). Mitral valve hemodynamic effects of percutaneous edge-to-edge repair with the MitraClip^TM^ device for mitral regurgitation. *Catheter. Cardiovasc. Interv.* 68 821–828. 10.1002/ccd.20917 17080467

[B27] Horstkotte J. Kloeser C. Beucher H. Schwarzlaender E. von Bardeleben R. S. Boekstegers P. (2016). Intraprocedural assessment of mitral regurgitation during the mitraclip procedure: impact of continuous left atrial pressure monitoring. *Catheter. Cardiovasc. Interv.* 88 1134–1143. 10.1002/ccd.26504 27038227

[B28] Hu Y. Shi L. Parameswaran S. Smirnov S. He Z. (2010). Left ventricular vortex under Mitral valve edge-to-edge repair. *Cardiovasc. Eng Techn.* 1 235–243. 10.1007/s13239-010-0022-6 21666755 PMC3110706

[B29] Jeyhani M. Shahriari S. Labrosse M. (2018). Experimental investigation of left ventricular flow patterns after Percutaneous Edge-to-Edge mitral valve repair. *Artif. Organs* 42 516–524. 10.1111/aor.13020 29168199

[B30] Jimenez J. H. Forbess J. Croft L. R. Small L. He Z. Yoganathan A. P. (2006). Effects of annular size, transmitral pressure, and mitral flow rate on the edge-to-edge repair: an in vitro study. *Ann. Thorac. Surg.* 82 1362–1368. 10.1016/j.athoracsur.2006.05.008 16996934

[B31] Kang W. S. Choi J. W. Kang J. E. Chung J. W. Kim S. H. (2013). Determination of mitral valve area with echocardiography, using intra-operative 3-dimensional versus intra- & post-operative pressure half-time technique in mitral valve repair surgery. *J. Cardiothor. Surg.* 8:98. 10.1186/1749-8090-8-98 23594408 PMC3642013

[B32] Kar S. Sharma R. (2015). Current assessment of mitral regurgitation: not making the grade. *J. Am. Coll. Cardiol.* 65 1089–1091. 10.1016/j.jacc.2015.02.001 25790879

[B33] Kong F. Caballero A. McKay R. Sun W. (2020). Finite element analysis of MitraClip procedure on a patient-specific model with functional mitral regurgitation. *J. Biomech.* 2020:109730. 10.1016/j.jbiomech.2020.109730 32147238

[B34] Kong F. Pham T. Martin C. Elefteriades J. McKay R. Primiano C. (2018). Finite element analysis of annuloplasty and papillary muscle relocation on a patient-specific mitral regurgitation model. *PLoS One* 13:e0198331. 10.1371/journal.pone.0198331 29902273 PMC6002124

[B35] Krieger E. V. Lee J. Branch K. R. Hamilton-Craig C. (2016). Quantitation of mitral regurgitation with cardiac magnetic resonance imaging: a systematic review. *Heart* 102 1864–1870. 10.1136/heartjnl-2015-309054 27733535

[B36] Kunzelman K. S. Quick D. W. Cochran R. P. (1998). Altered collagen concentration in mitral valve leaflets: biochemical and finite element analysis. *Ann. Thorac. Surg.* 66 (6 Suppl), S198–S205. 10.1016/s0003-4975(98)01106-0 9930448

[B37] Kuwata S. Taramasso M. Czopak A. Luciani M. Pozzoli A. Ho E. (2019). Continuous direct left atrial pressure: intraprocedural measurement predicts clinical response following MitraClip therapy. *JACC Cardiovasc. Interv.* 12 127–136. 10.1016/j.jcin.2018.07.051 30594511

[B38] Ladich E. Michaels M. B. Jones R. M. McDermott E. Coleman L. Komtebedde J. (2011). Endovascular Valve Edge-to-Edge Repair Study I. Pathological healing response of explanted MitraClip devices. *Circulation* 123 1418–1427. 10.1161/CIRCULATIONAHA.110.978130 21422390

[B39] Latib A. Ancona M. B. Ferri L. Montorfano M. Mangieri A. Regazzoli D. (2016). Percutaneous direct annuloplasty with cardioband to treat recurrent mitral regurgitation after MitraClip implantation. *JACC Cardiovasc. Interv.* 9 e191–e192. 10.1016/j.jcin.2016.06.028 27592014

[B40] Lau K. Diaz V. Scambler P. Burriesci G. (2010). Mitral valve dynamics in structural and fluid–structure interaction models. *Med. Eng. Phys.* 32 1057–1064. 10.1016/j.medengphy.2010.07.008 20702128 PMC2989441

[B41] Lau K. Díaz-Zuccarini V. Scambler P. Burriesci G. (2011). Fluid–structure interaction study of the edge-to-edge repair technique on the mitral valve. *J. Biomech.* 44 2409–2417. 10.1016/j.jbiomech.2011.06.030 21767842

[B42] Lim D. S. Reynolds M. R. Feldman T. Kar S. Herrmann H. C. Wang A. (2014). Improved functional status and quality of life in prohibitive surgical risk patients with degenerative mitral regurgitation after transcatheter mitral valve repair. *J. Am. Coll. Cardiol.* 64 182–192. 10.1016/j.jacc.2013.10.021 24184254

[B43] Liu M. Liang L. Sun W. (2017). A new inverse method for estimation of in vivo mechanical properties of the aortic wall. *J. Mech. Behav. Biomed. Mater.* 72 148–158. 10.1016/j.jmbbm.2017.05.001 28494272 PMC5525022

[B44] Magruder J. T. Crawford T. C. Grimm J. C. Fredi J. L. Shah A. S. (2016). Managing mitral regurgitation: focus on the MitraClip device. *Med. Devices* 9 53–60. 10.2147/MDER.S86645 27110142 PMC4835144

[B45] Mahmood F. Gorman J. H. III Subramaniam B. Gorman R. C. Panzica P. J. Hagberg R. C. (2010). Changes in mitral valve annular geometry after repair: saddle-shaped versus flat annuloplasty rings. *Ann. Thorac. Surg.* 90 1212–1220. 10.1016/j.athoracsur.2010.03.119 20868816 PMC3021250

[B46] Mansi T. Voigt I. Georgescu B. Zheng X. Mengue E. A. Hackl M. (2012). An integrated framework for finite-element modeling of mitral valve biomechanics from medical images: application to MitralClip intervention planning. *Med. Image Anal.* 16 1330–1346. 10.1016/j.media.2012.05.009 22766456

[B47] Mantegazza V. Pasquini A. Agati L. Fusini L. Muratori M. Gripari P. (2018). Comprehensive assessment of mitral valve geometry and cardiac remodeling with 3-dimensional echocardiography after Percutaneous Mitral valve repair. *Am. J. Cardiol.* 122 1195–1203. 10.1016/j.amjcard.2018.06.036 30082038

[B48] Mao W. Caballero A. Hahn R. T. Sun W. (2020). Comparative quantification of primary mitral regurgitation by computer modeling and simulated echocardiography. *Am. J. Physiol. Heart Circ. Physiol.* 318 H547–H557. 10.1152/ajpheart.00367.2019 31922890 PMC7099454

[B49] Mao W. Caballero A. McKay R. Primiano C. Sun W. (2017). Fully-coupled fluid-structure interaction simulation of the aortic and mitral valves in a realistic 3D left ventricle model. *PLoS One* 12:e0184729. 10.1371/journal.pone.0184729 28886196 PMC5590990

[B50] Mao W. B. Li K. W. Caballero A. Sun W. (2016a). Fully-Coupled FSI simulation of bioprosthetic heart valve using smoothed particle hydrodynamics. *Cardiology* 134:178. 10.1007/s13239-016-0285-7 27844463 PMC5289304

[B51] Mao W. B. Li K. Sun W. (2016b). Fluid–structure interaction study of transcatheter aortic valve dynamics using smoothed particle hydrodynamics. *Cardiovasc. Eng. Technol.* 7 374–388. 10.1007/s13239-016-0285-7 27844463 PMC5289304

[B52] Mauri L. Foster E. Glower D. D. Apruzzese P. Massaro J. M. Herrmann H. C. (2013). 4-year results of a randomized controlled trial of percutaneous repair versus surgery for mitral regurgitation. *J. Am. Coll. Cardiol.* 62 317–328. 10.1016/j.jacc.2013.04.030 23665364

[B53] McCarthy K. P. Ring L. Rana B. S. (2010). Anatomy of the mitral valve: understanding the mitral valve complex in mitral regurgitation. *Eur. J. Echocardiogr.* 11 i3–i9. 10.1093/ejechocard/jeq153 21078837

[B54] Mokadam N. A. Stout K. K. Verrier E. D. (2011). Management of acute regurgitation in left-sided cardiac valves. *Tex. Heart Inst. J.* 38 9–19. 21423463 PMC3060740

[B55] Morgan A. E. Wozniak C. J. Gulati S. Ge L. Grossi E. A. Weinsaft J. W. (2017). Uneven MitraClip application does not increase leaflet stress in a finite element model. *JAMA Surg.* 152:111. 10.1001/jamasurg.2016.3360 27706490 PMC5453713

[B56] Neuss M. Schau T. Isotani A. Pilz M. Schopp M. Butter C. (2017). Elevated Mitral Valve pressure gradient after MitraClip implantation deteriorates long-term outcome in patients with severe Mitral regurgitation and severe heart failure. *JACC Cardiovasc. Interv.* 10 931–939. 10.1016/j.jcin.2016.12.280 28473116

[B57] Noack T. Janietz M. Lurz P. Kiefer P. Sieg F. Marin-Cuartas M. (2019a). Dynamic mitral valve geometry in patients with primary and secondary mitral regurgitation: implications for mitral valve repair. *Eur. J. Cardiothorac. Surg.* 56 983–992. 10.1093/ejcts/ezz096 30932164

[B58] Noack T. Kiefer P. Mallon L. Lurz P. Bevilacqua C. Banusch J. (2019b). Changes in dynamic mitral valve geometry during Percutaneous edge–edge mitral valve repair with the MitraClip system. *J. Echocardiogr.* 17 84–94. 10.1007/s12574-018-0398-0 30291509

[B59] Obadia J. F. Messika-Zeitoun D. Leurent G. Iung B. Bonnet G. Piriou N. (2018). Percutaneous repair or medical treatment for secondary mitral regurgitation. *N. Engl. J. Med.* 379 2297–2306. 10.1056/NEJMoa1805374 30145927

[B60] Palmiero G. Ascione L. Briguori C. Carlomagno G. Sordelli C. Ascione R. (2017). The mitral-to-aortic flow-velocity integral ratio in the real world echocardiographic evaluation of functional mitral regurgitation before and after percutaneous repair. *J. Interv. Cardiol.* 30 368–373. 10.1111/joic.12401 28675000

[B61] Paranskaya L. D’Ancona G. Bozdag-Turan I. Akin I. Kische S. Turan G. R. (2013). Residual mitral valve regurgitation after percutaneous mitral valve repair with the mitraclip^®^ system is a risk factor for adverse one-year outcome. *Catheter. Cardiovasc. Interv.* 81 609–617. 10.1002/ccd.24586 22887450

[B62] Patzelt J. Zhang Y. Magunia H. Ulrich M. Jorbenadze R. Droppa M. (2017). Improved mitral valve coaptation and reduced mitral valve annular size after percutaneous mitral valve repair (PMVR) using the MitraClip system. *Eur. Heart J. Cardiovasc. Imaging* 19 785–791. 10.1093/ehjci/jex173 28977372

[B63] Pham T. Kong F. Martin C. Wang Q. Primiano C. McKay R. (2017). Finite element analysis of patient-specific mitral valve with mitral regurgitation. *Cardiovasc. Eng. Technol.* 8 3–16. 10.1007/s13239-016-0291-9 28070866 PMC5321865

[B64] Prescott B. Abunassar C. Baxevanakis K. P. Zhao L. (2019). Computational evaluation of mitral valve repair with MitraClip^∗^.

[B65] Quick D. W. Kunzelman K. S. Kneebone J. M. Cochran R. P. (1997). Collagen synthesis is upregulated in mitral valves subjected to altered stress. *ASAIO J.* 43 181–186. 9152488

[B66] Redaelli A. Guadagni G. Fumero R. Maisano F. Alfieri O. (2001). A computational study of the hemodynamics after “edge-to-edge” mitral valve repair. *J. Biomech. Eng.* 123 565–570. 10.1115/1.1408938 11783727

[B67] Rogers J. H. Boyd W. D. Smith T. W. R. Ebner A. A. Bolling S. F. (2018). Combined MitraClip edge-to-edge repair with millipede IRIS Mitral annuloplasty. *JACC Cardiovasc. Interv.* 11 323–324. 10.1016/j.jcin.2017.11.007 29413250

[B68] Sacks M. Drach A. Lee C. H. Khalighi A. Rego B. Zhang W. (2019). On the simulation of mitral valve function in health, disease, and treatment. *J. Biomech. Eng.* 141:070804. 10.1115/1.4043552 31004145 PMC6611349

[B69] Saikrishnan N. Kumar G. Sawaya F. J. Lerakis S. Yoganathan A. P. (2014). Accurate assessment of aortic stenosis: a review of diagnostic modalities and hemodynamics. *Circulation* 129 244–253. 10.1161/CIRCULATIONAHA.113.002310 24421359

[B70] Schmidt F. P. von Bardeleben R. S. Nikolai P. Jabs A. Wunderlich N. Münzel T. (2013). Immediate effect of the MitraClip^®^ procedure on mitral ring geometry in primary and secondary mitral regurgitation. *Eur. Heart J. Cardiovasc. Imaging* 14 851–857. 10.1093/ehjci/jes293 23288891

[B71] Schueler R. Kaplan S. Melzer C. Ozturk C. Weber M. Sinning J. M. (2017). Impact of interventional edge-to-edge repair on mitral valve geometry. *Int. J. Cardiol.* 230 468–475. 10.1016/j.ijcard.2016.12.081 28041699

[B72] Schueler R. Momcilovic D. Weber M. Welz A. Werner N. Mueller C. (2014). Acute changes of mitral valve geometry during interventional edge-to-edge repair with the MitraClip system are associated with midterm outcomes in patients with functional valve disease: preliminary results from a prospective single-center study. *Circ. Cardiovasc. Interv.* 7 390–399. 10.1161/CIRCINTERVENTIONS.113.001098 24895448

[B73] Shi L. He Z. (2009). Hemodynamics of the mitral valve under edge-to-edge repair: an in vitro steady flow study. *J. Biomech. Eng.* 131:51010. 10.1115/1.3118772 19388780

[B74] Singh G. D. Smith T. W. Rogers J. H. (2015). Multi-M itra C lip therapy for severe degenerative mitral regurgitation:“Anchor” technique for extremely flail segments. *Catheter. Cardiovasc. Interv.* 86 339–346. 10.1002/ccd.25811 25559345

[B75] Sorajja P. Vemulapalli S. Feldman T. Mack M. Holmes D. R. Jr. Stebbins A. (2017). Outcomes with transcatheter Mitral valve repair in the United States: an STS/ACC TVT registry report. *J. Am. Coll. Cardiol.* 70 2315–2327. 10.1016/j.jacc.2017.09.015 29096801

[B76] Stone G. W. Lindenfeld J. Abraham W. T. Kar S. Lim D. S. Mishell J. M. (2018). Transcatheter mitral-valve repair in patients with heart failure. *N. Engl. J. Med.* 379 2307–2318. 10.1056/NEJMoa1806640 30280640

[B77] Stone G. W. Vahanian A. S. Adams D. H. Abraham W. T. Borer J. S. Bax J. J. (2015). Clinical trial design principles and endpoint definitions for Transcatheter Mitral valve repair and replacement: part 1: clinical trial design principles a consensus document from the Mitral Valve Academic Research Consortium. *J. Am. Coll. Cardiol.* 66 278–307. 10.1016/j.jacc.2015.05.046 26184622

[B78] Sturla F. Redaelli A. Puppini G. Onorati F. Faggian G. Votta E. (2015). Functional and biomechanical effects of the Edge-to-Edge Repair in the Setting of Mitral regurgitation: consolidated knowledge and novel tools to gain insight into its Percutaneous implementation. *Cardiovasc. Eng. Technol.* 6 117–140. 10.1007/s13239-014-0208-4 26577231

[B79] Sturla F. Vismara R. Jaworek M. Votta E. Romitelli P. Pappalardo O. A. (2017). In vitro and in silico approaches to quantify the effects of the Mitraclip^®^ system on mitral valve function. *J. Biomech.* 50 83–92. 10.1016/j.jbiomech.2016.11.013 27863743

[B80] Turyan Medvedovsky A. Tonchev I. Tahiroglu I. Lotan C. Gilon D. Planer D. (2019). MitraClip therapy in critically Ill patients with severe functional mitral regurgitation and refractory heart failure. *Struct. Heart* 2019 1–6.

[B81] Wang Q. Primiano C. Sun W. (2014). Can isolated annular dilatation cause significant ischemic mitral regurgitation? Another look at the causative mechanisms. *J. Biomech.* 47 1792–1799. 10.1016/j.jbiomech.2014.03.033 24767703

[B82] Wang Q. Sun W. (2013). Finite element modeling of mitral valve dynamic deformation using patient-specific multi-slices computed tomography scans. *Ann. Biomed. Eng.* 41 142–153. 10.1007/s10439-012-0620-6 22805982

[B83] Warraich H. J. Chaudary B. Maslow A. Panzica P. J. Pugsley J. Mahmood F. (2012). Mitral annular nonplanarity: correlation between annular height/commissural width ratio and the nonplanarity angle. *J. Cardiothorac. Vasc. Anesth.* 26 186–190. 10.1053/j.jvca.2011.09.007 22051418

[B84] Zhang Y. Wang V. Y. Morgan A. E. Kim J. Handschumacher M. D. Moskowitz C. S. (2019). Mechanical effects of MitraClip on leaflet stress and myocardial strain in functional mitral regurgitation–A finite element modeling study. *PLoS One* 14:e0223472. 10.1371/journal.pone.0223472 31600276 PMC6786765

[B85] Zhong Q. Zeng W. Huang X. Zhao X. (2014). Finite element analysis for edge-to-edge technique to treat post-mitral valve repair systolic anterior motion. *Acta Bioeng. Biomech.* 16 3–12. 25597231

[B86] Zoghbi W. A. Adams D. Bonow R. O. Enriquez-Sarano M. Foster E. Grayburn P. A. (2017). Recommendations for noninvasive evaluation of native valvular regurgitation: a report from the American Society of echocardiography developed in collaboration with the society for cardiovascular Magnetic resonance. *J. Am. Soc. Echocardiogr.* 30 303–371. 10.1016/j.echo.2017.01.007 28314623

[B87] Zoghbi W. A. Chambers J. B. Dumesnil J. G. Foster E. Gottdiener J. S. Grayburn P. A. (2009). Recommendations for Evaluation of Prosthetic Valves With Echocardiography and Doppler Ultrasound: a Report From the American Society of Echocardiography’s Guidelines and Standards Committee and the Task Force on Prosthetic Valves, Developed in Conjunction With the American College of Cardiology Cardiovascular Imaging Committee, Cardiac Imaging Committee of the American Heart Association, the European Association of Echocardiography, a Registered Branch of the European Society of Cardiology, the Japanese Society of Echocardiography and the Canadian Society of Echocardiography, Endorsed by the American College of Cardiology Foundation, American Heart Association, European Association of Echocardiography, a Registered Branch of the European Society of Cardiology, the Japanese Society of Echocardiography, and Canadian Society of Echocardiography. *J Am Soc Echocardiogr.* 22 975–1014. 10.1016/j.echo.2009.07.013 19733789

[B88] Zoghbi W. A. Enriquez-Sarano M. Foster E. Grayburn P. A. Kraft C. D. Levine R. A. (2003). Recommendations for evaluation of the severity of native valvular regurgitation with two-dimensional and Doppler echocardiography. *J. Am. Soc. Echocardiogr* 16 777–802. 10.1016/S0894-7317(03)00335-3 12835667

