# Supplementary material for: A Comprehensive Engineering Analysis of Left Heart Dynamics After MitraClip in a Functional Mitral Regurgitation Patient
Source: Front Physiol. 2020 May 7;11:432. doi: 10.3389/fphys.2020.00432 (PMC7221026; doi:10.3389/fphys.2020.00432)
Supplement: Supplementary file 1 [file Table_1.DOCX]

**Supplementary Material**

**Patient-specific LH model with functional MR**

The MSCT images, acquired with a GE LightSpeed 64-channel volume CT scanner, had an in-plane resolution of 0.82 x 0.82 mm and a slice thickness of 0.625 mm. Ten phases of the cardiac cycle were collected using an ECG-gated sequence. DICOM images were imported into Amira-Avizo (Thermo Fisher Scientific, MA) and 3D Slicer (www.slicer.org) software to segment the cardiac structures. HyperMesh software (Altair Engineering, Inc., MI) was then used to create a high quality FE mesh. The AV and MV geometries were segmented at mid-systole and mid-diastole, respectively, approximating their stress–free configuration ^1^. The patient-specific MV model used in this study was developed and validated in a previous work from our group that investigated MV dynamics under functional MR ^2^. Briefly, the detailed chordae structure (number, position, length, branching, origins of the PM tips, and insertions into the leaflets) was directly reconstructed from the MSCT images. Chordae were classified into five groups: anterior strut, anterior basal, anterior marginal, posterior basal, and posterior marginal. Cross-sectional area values of 0.38 mm^2^, 0.71 mm^2^ and 2.05 mm^2^ were assigned to marginal, basal and strut chordae, respectively ^3^. A total of 18 chordae origins were modeled from the PM tips.

3D solid elements (eight-node hexahedral C3D8R/C3D8I elements, six-node wedge C3D6 elements, and four-node tetrahedral C3D4 elements) were used to discretize the ascending aorta, aortic root, AV, calcification and MV. Stress/displacement truss elements (two-node linear T3D2 elements) were used for the mitral chordae, while shell elements (four-node quadrilateral S4 elements) were used to model the endocardial wall. Two layers of elements were used across the mitral leaflet thickness. For the AML, the average thickness values for the leaflet belly and free edge were 1.26 mm and 2.09 mm, respectively. For the PML, the average thickness values of the leaflet belly and free edge were 1.31 mm and 1.57 mm, respectively ^2^. Four layers of elements were used across the ascending aorta/aortic root and AV leaflet thickness, with a uniform total thickness of 2 mm and 0.7 mm, respectively.

Cardiac tissues were assumed to be homogeneous, non-linear and elastic. An anisotropic hyperelastic material model (MHGO) based on Holzapfel et al. ^4,5^ was adopted to characterize the mechanical response of the ascending aorta, aortic root, AV leaflets, MV leaflets, and myocardium. The strain energy function of the MHGO model, $W$, is given by Equation 1:

$W=C_{10}\left\{ \exp\left[ C_{01}\left( \bar{I}_{1}-3 \right) \right]-1 \right\}+\frac{k_{1}}{2k_{2}}\sum_{i=1}^{2} \left[ \exp k_{2}\left[ \kappa\bar{I}_{1}+\left( 1-3\kappa\right)\bar{I}_{4_{i}}-1 \right]^{2}-1 \right]+\frac{1}{D}{(J-1)}^{2}, i=1,2$ (1)

where $C_{10}$, $C_{01}$, $k_{1}$, $k_{2}$, $\kappa$ and $D$ are material constants, and $\bar{I}_{1}$and $\bar{I}_{4i}$are the deviatoric strain invariants. $C_{10}$ and $C_{01}$ describe the matrix material, $D$ is a material constant to impose incompressibility, and $J$ is the determinant of the deformation gradient. $k_{1}$ is a positive constant with the dimension of stress to describe the fiber material and $k_{2}$ is a dimensionless parameter. In addition, $\kappa$ describes the distribution of fiber orientation. Local coordinate systems were defined for each cardiac tissue to include local fiber orientation. The MHGO material model was implemented into Abaqus 6.17/Explicit (Dassault Systèmes Simulia Corp., Providence, RI, USA) with a user sub-routine VUMAT. Additionally, the isotropic hyperelastic Ogden material model ^6^ was used to characterize the mechanical behavior of mitral chordae and the aortic-mitral curtain. The Ogden strain energy function is given by Equation 2:

|  | $W=\sum_{i=1}^{N} \frac{2\mu_{i}}{a_{i}^{2}}(\bar{\lambda}_{1}^{a_{i}}+\bar{\lambda}_{2}^{a_{i}}+\bar{\lambda}_{3}^{a_{i}}-3)$ | (2) |
| --- | --- | --- |

where $\mu_{i}$ and $a_{i}$ are material constants, and $\bar{\lambda}_{i}$ are the modified principal stretches. In-house multiprotocol biaxial and uniaxial testing data of human cardiac tissues were used to obtain the material properties selected from an existing human cardiac tissue database established in our lab (age- and gender-matched patient), as listed in Supplementary Table 1.

**Supplementary Table 1.** LH tissue material parameters

| **MHGO model** | $C_{10}$($\mathrm{kPa}$) | $C_{01}$ | $k_{1}$ ($\mathrm{kPa}$) | $k_{2}$ | $\theta(^{\circ})$ | $\kappa$ | $D (\mathrm{kP}a^{-1})$ |
| --- | --- | --- | --- | --- | --- | --- | --- |
| AV leaflets | 0.017 | 147.262 | 39704.1 | 2352.96 | 0 | 0.317 | 5.0e-4 |
| AML | 0.285 | 61.303 | 9.295 | 99.684 | 0 | 0.333 | 5.0e-4 |
| PML | 0.101 | 33.191 | 10.756 | 48.495 | 27.98 | 0.089 | 5.0e-4 |
| Root | 1.755 | 13.707 | 10.550 | 80.379 | 20.06 | 0 | 5.0e-4 |
| Aorta | 4.175 | 3.464 | 3.771 | 15.927 | 70.95 | 0.086 | 5.0e-4 |
| Myocardium | 0.0374 | 15.387 | 6.079 | 98.366 | 6.78 | 0.144 | 5.0e-4 |
| **Ogden model** | $\mu_{1}$($\mathrm{MPa}$) | $a_{1}$ | $\mu_{2}$($M\mathrm{Pa}$) | $a_{2}$ | $\mu_{3}$($\mathrm{MPa}$) | $a_{3}$ |  |
| Anterior marginal | 17.824 | 17.808 | 17.660 | 17.797 | 17.592 | 17.768 |  |
| Anterior strut | 24.342 | 11.338 | 10.332 | 11.167 | 14.914 | 11.188 |  |
| Anterior/posterior basal | 10.256 | 16.579 | 10.654 | 16.554 | 10.671 | 16.554 |  |
| Posterior marginal | 12.995 | 15.651 | 13.083 | 15.683 | 12.870 | 15.662 |  |
| Aortic-mitral curtain | 1.505 | 21.400 | 11.207 | 21.400 | 1.441 | 21.400 |  |

**Validation studies**

Two previous computer studies have been conducted for the validation of the patient-specific LH model used in this investigation. The first study evaluated the accuracy of the structural MV model with functional MR by quantitatively comparing the closed MV geometry obtained from the simulation with the in vivo MV geometry obtained from the MSCT images at systole. MV model optimization, including chordae tethering adjustment and pretension were performed until correct valve morphology and leaflet deformation were obtained ^2^. The second study focused on the accuracy of the FSI modeling framework in simulating patient-specific LH hemodynamics before and after TAVR. Comparison of FSI results with the patient’s available pre- and post-operative echo measurements revealed a close quantitative agreement in terms of major hemodynamic variables such as transvalvular pressure gradients, blood velocities, stroke and regurgitant volumes, valve orifice area, etc ^7^. Overall, these studies demonstrated the robustness and the predictive capabilities of our SPH-FE FSI modeling framework in simulating pre- and post-procedure patient-specific LH dynamics.

**Supplementary References**

1 Sun, W., Martin, C. & Pham, T. Computational modeling of cardiac valve function and intervention. *Annual review of biomedical engineering* **16**, 53-76 (2014).

2 Pham, T. *et al.* Finite Element Analysis of Patient-Specific Mitral Valve with Mitral Regurgitation. *Cardiovascular engineering and technology* **8**, 3-16 (2017).

3 Wang, Q. & Sun, W. Finite element modeling of mitral valve dynamic deformation using patient-specific multi-slices computed tomography scans. *Annals of biomedical engineering* **41**, 142-153 (2013).

4 Holzapfel, G. A., Gasser, T. C. & Ogden, R. W. A New Constitutive Framework for Arterial Wall Mechanics and a Comparative Study of Material Models. *Journal of elasticity and the physical science of solids* **61**, 1-48, doi:10.1023/a:1010835316564 (2000).

5 Gasser, T. C., Ogden, R. W. & Holzapfel, G. A. Hyperelastic modelling of arterial layers with distributed collagen fibre orientations. *Journal of The Royal Society Interface* **3**, 15-35, doi:10.1098/rsif.2005.0073 (2006).

6 Ogden, R. W. Large Deformation Isotropic Elasticity - On the Correlation of Theory and Experiment for Incompressible Rubberlike Solids. *Proceedings of the Royal Society of London. A. Mathematical and Physical Sciences* **326**, 565-584, doi:10.1098/rspa.1972.0026 (1972).

7 Caballero, A., Mao, W., McKay, R. & Sun, W. The impact of balloon-expandable transcatheter aortic valve replacement on concomitant mitral regurgitation: a comprehensive computational analysis. *J R Soc Interface* **16**, 20190355, doi:10.1098/rsif.2019.0355 (2019).
